# From the epidemiology of hepatitis E virus (HEV) within the swine reservoir to public health risk mitigation strategies: a comprehensive review

**DOI:** 10.1186/s13567-017-0436-3

**Published:** 2017-05-25

**Authors:** Morgane Salines, Mathieu Andraud, Nicolas Rose

**Affiliations:** 1 ANSES-Ploufragan-Plouzané Laboratory, BP 53, 22440 Ploufragan, France; 2 Université Bretagne Loire, Rennes, France

## Abstract

Hepatitis E virus (HEV) is the causative agent of hepatitis E in humans, an emerging zoonosis mainly transmitted via food in developed countries and for which domestic pigs are recognised as the main reservoir. It therefore appears important to understand the features and drivers of HEV infection dynamics on pig farms in order to implement HEV surveillance programmes and to assess and manage public health risks. The authors have reviewed the international scientific literature on the epidemiological characteristics of HEV in swine populations. Although prevalence estimates differed greatly from one study to another, all consistently reported high variability between farms, suggesting the existence of multifactorial conditions related to infection and within-farm transmission of the virus. Longitudinal studies and experimental trials have provided estimates of epidemiological parameters governing the transmission process (e.g. age at infection, transmission parameters, shedding period duration or lag time before the onset of an immune response). Farming practices, passive immunity and co-infection with immunosuppressive agents were identified as the main factors influencing HEV infection dynamics, but further investigations are needed to clarify the different HEV infection patterns observed in pig herds as well as HEV transmission between farms. Relevant surveillance programmes and control measures from farm to fork also have to be fostered to reduce the prevalence of contaminated pork products entering the food chain.

**Electronic supplementary material:**

The online version of this article (doi:10.1186/s13567-017-0436-3) contains supplementary material, which is available to authorized users.

## Introduction

Hepatitis E virus (HEV) is a non-enveloped single-stranded RNA virus. It is transmitted via the faecal-oral route and causes acute hepatitis in humans, clinical signs being similar to hepatitis A infection but usually more severe [1]. Chronic cases have also been described in immunocompromised patients [2]. There are four HEV genotypes: genotypes 1 and 2 are specific to humans and are currently circulating in Asia, Africa and Central America in epidemic waves linked to the consumption of contaminated water [3]. Genotypes 3 and 4 are responsible for sporadic autochthonous human cases in developed countries and are common to humans and other animal species [3, 4]. Genotype 3 in particular is highly prevalent in wild and domestic pigs, but the infection does not lead to a clinical disease [5]. Swine and human HEV strains are genetically very close, and cross-species transmission has been proved [6]. Moreover, a number of sporadic autochthonous cases have been related to the consumption of raw or undercooked pork products, especially liver-based products [7–9]. Thus, hepatitis E is considered to be an emerging zoonosis, domestic pigs being recognised as its main reservoir in industrialised countries [4, 10]. It is crucial to fully understand the conditions related to pig farm infection and HEV transmission dynamics within the swine population in order to limit the risk of introducing contaminated products into the food chain.

Several prevalence studies have been carried out in pig herds, either on a farm or individual scale. Prevalence estimates derived from either virological or serological analyses have evidenced wide differences depending on the country and year of study. However, the available data are difficult to compare since the pigs’ age and production stage vary according to studies, as do the HEV detection methods and biological matrix used for analyses. Moreover, the precision of the different estimates varies greatly between studies owing to huge differences in sample sizes. Even within the same study, the individual and farm-scale prevalences observed are also highly heterogeneous. This wide dispersion suggests the existence of various infection dynamics linked to farm-specific risk factors which have only been sporadically investigated to date. Observational studies mainly report the implication of farming practices in terms of hygiene, biosecurity and rearing conditions. Complementary to this approach, mathematical modelling studies, based either on experimental trials or longitudinal studies on infected farms have helped reveal new insights on HEV infection dynamics.

It is important to explore the epidemiological characteristics of HEV on pig farms for several purposes, e.g. to set up a surveillance programme, or identify control measures to manage the risk of HEV infection and transmission with the ultimate aim of reducing the prevalence of HEV-containing livers at the slaughterhouse. Information available from published papers has therefore been comprehensively gathered to identify key patterns of HEV infection as well as knowledge gaps and research needs. We have specifically focused our study on the epidemiological characteristics of the virus in domestic pigs and their products, since other aspects of this zoonosis have already been reviewed in various papers. The scope of this review thus covers prevalence, risk factors, transmission routes and infection dynamics on pig farms, surveillance and control strategies throughout the pork chain.

## HEV prevalence in farmed pigs

It is crucial to know the prevalence of HEV on pig farms so as to be able to assess the health situation of the pig population and thus the risk to public health. We counted 86 studies (from 43 different countries) addressing HEV prevalence in farmed pigs. These studies are summarised in Additional file 1 [5, 11–21, 24, 47, 57, 66, 70–73, 79–139]. Various methods were used for data collection: samples were collected from slaughterhouses, randomly-selected or specifically-selected farms, or from serum/faeces/organ banks. Some studies were conducted at a given point in time, leading to an instantaneous prevalence estimate, whereas others were retrospective and estimated the prevalence from sera collected over a given period of time. The number of farms varied from 1 to 2 001; the number of samples from 40 to 6 565. Pigs included in the studies differed in age and rearing conditions (family-scale or large-scale farms, organic or industrial production, for example). Little information was available on the swine breed or strain. Prevalence was investigated either on a farm or individual level. The virus was sought in several different biological materials, including various organs (e.g. the intestines or liver), serum, faeces, bile and caecal content. Individual or pooled samples were processed using RT-PCR, nested RT-PCR, real-time RT-PCR or antigen detection. The serological response to HEV infection was assessed by detection of anti-HEV antibodies (IgG, IgM or IgA) using ELISA tests with specificity ranging from 85 to 100% and sensitivity from 50 to 100%. The viral strains detected belonged either to genotype 3 or genotype 4.

### Farm-scale prevalence

#### Farm-scale seroprevalence

Farm-scale seroprevalence reported in 15 studies ranged from 30 to 98% (Additional file 1). For instance, in a study conducted in France in 2011, 65% of the 186 randomly-selected farms were found to have at least one seropositive animal (95% confidence interval 57–74) [5]. The serological prevalence was even higher in a retrospective study conducted in Spain, 204 out of 208 farms (98%, 95% CI 96.1–99.9) having at least one anti-HEV IgG-positive pig [11]. Similarly, in a retrospective study recently carried out in Norway, anti-HEV IgG were detected in 90% (137/153) of the herds [12].

#### Farm-scale virological prevalence

Farm-scale virological prevalence reported in 25 studies ranged from 10 to 100% (Additional file 1). Widen et al. detected HEV-RNA in swine faeces from 17 out of 22 randomly-selected farms in Sweden (72.7%) [13]. Virological prevalence has also been estimated from HEV-RNA detection in sera: in 72 herds selected in Spain, at least one slaughtered pig tested positive for HEV-RNA in serum on 47.2% of farms [14]. Regarding the presence of HEV RNA in liver, 24% (95% CI 17–31) of 186 randomly-selected pig farms had at least one positive liver in the French national prevalence study conducted by Rose et al. in 2011 [5].

### Individual prevalence

#### Individual seroprevalence

Individual seroprevalence ranged from 8 to 93% in the 45 studies analysed (Additional file 1). In France, 31% (95% CI 24–38) of the slaughter-aged pigs in 2011 were found HEV seropositive [5]. Similarly, Jinshan et al. detected 52% of sampled pigs positive for anti-HEV antibodies in Mongolia [15]. Crossan et al. separately tested the presence of the different types of anti-HEV antibodies in Scotland and reported that, of 176 serum samples tested, 29% (*n* = 51) were anti-HEV IgG-positive, 36.9% (*n* = 65) anti-HEV IgA-positive and 29% (*n* = 51) anti-HEV IgM-positive. Overall seroprevalence (anti-HEV IgG+ and/or IgA+ and/or IgM+) was 61.4% (*n* = 108) [16]. In the same region and period, individual HEV seroprevalence was found by Grierson et al. to be even higher; they reported that 584 out of 629 pigs (92.8%) had anti-HEV antibodies at the time of slaughter [17].

#### Individual virological prevalence

Individual virological prevalence ranged from 1 to 89% in the 69 reported studies (Additional file 1). For instance, the HEV genome was detected in the faeces of 42% of 274 randomly-selected pigs from six different swine farms in northern Italy [18]. HEV RNA was also detected in serum: Crossan et al. reported a virological prevalence of 44.4% in serum (72/162) [16], whereas Grierson et al. detected HEV RNA in only 3% of plasma samples (22/629) in pigs at slaughter age [17]. In the same study, 15% of caecal contents (93/629) were found positive to HEV RNA [17]. Regarding the detection of HEV in liver, Rose et al. reported an individual prevalence of HEV RNA-positive livers of 4% (95% CI 2–6) at slaughter age [5].

Both at farm and individual levels, studies carried out in a given country at different times or retrospectively did not show any significant change in prevalence estimates over time, suggesting that HEV was constantly circulating in pig farms. The marked variability in individual prevalence estimates between farms is noteworthy: from 12.8 to 72.5% in Italy [18], from 4 to 58% in Argentina [19], and from 5 to 90% in France [5]. This may reflect different infection dynamics related to farm-specific risk factors.

### Factors influencing HEV prevalence estimates

To date, few studies have reported the risk factors associated with high HEV prevalence on pig farms. We identified 12 studies addressing HEV risk factors, but only six of them quantified the impact of risk factors on HEV seroprevalence or on the prevalence of shedding pigs through odds ratio estimates (Table 1). The risk factors for a high HEV seroprevalence were mainly related to (1) farm characteristics and (2) farming practices. The farming scale (medium-size and family-scale farms, linked to the number of pigs and sows) was identified as a risk factor related to HEV seroprevalence [15, 18, 20, 21]. It was also shown that HEV seroprevalence was significantly higher in organic farms than in conventional ones [22]. Several high-risk rearing practices were reported, the main ones being late weaning, mingling practices at the nursery stage and poor hygiene [23]. Biosecurity measures such as requiring a shower upon entry were also found to be protective factors with respect to the prevalence of faecal HEV RNA shedding [24]. A seasonal influence on the prevalence of HEV RNA among swine was also reported, with a major peak in March–April followed by a smaller peak in September–October [25].

**Table 1:** **Quantified risk factors associated with a high HEV seroprevalence in pig farms**

Di Bartolo et al. [18]	Number of sows > 1000: HEV seroprevalence = 54.2 vs 18.9%
Li et al. [21]	HEV seroprevalence on family-scale farms = 90 vs 76% in large-scale farms (*p* < 0.01)
Jinshan et al. [15]	Number of pigs > 600: HEV seroprevalence ranged from 78 to 100%, vs 0 to 29%
Hinjoy et al. [20]	Medium-sized farms compared with large farms: OR 4.95 (1.79–13.70)
	Presence of bird faeces inside the pig house: OR 2.87 (1.07–7.71)
Walachowski et al. [23]	Duration of the nursery down period < 4 days: OR 1.7 (1.04–2.9)
	Distance between pit manure and slatted floor in fattening premises < 80 cm: OR 1.9 (1.1–3.5)
	Mingling of pigs from different premises between farrowing and nursery stages: OR 1.8 (1.1–2.9)
	Pen size in nursery rooms > 26 pigs/pen: OR 2.4 (1.2–4.8)
Rutjes et al. [22]	HEV seroprevalence on organic farms = 89 vs 72% on conventional farms (*p* = 0.04)
	HEV seroprevalence on free-range farms = 76 vs 72% on conventional farms (*p* = 0.06)

*OR* odds ratio.

European wild boars are recognized as a potential reservoir of HEV [26, 27]. Moreover, some experimental studies evidenced that HEV strains could be transmitted from European wild boars (*Sus scrofa*) to domestic pigs [26, 28, 29]. Though no study directly related HEV prevalence in pig farms to contact with wild boars, they may play a potential role in the swine HEV epidemiology in free-ranged pig production units. The role of wild boars as HEV risk exposure for domestic pigs would deserve further investigation.

## HEV infection characteristics and dynamics on pig farms

### HEV infection features in pigs

#### Age at HEV infection and shedding

The age at infection was only sporadically reported in the literature, with only three studies inferring from serological results the window within which infection took place. Almost all the studies conducted on pig farms only reported the age at shedding, and not the age at infection. Based on a large-scale seroprevalence survey conducted in Japan, the average age at infection was estimated to range from 59.0 to 67.3 days with more than 80% of infections occurring between the ages of 30 and 90 days [30]. The results of a longitudinal study on three French farms were quite different, most HEV infections occurring between 105 and 140 days of age [31]. Using Spanish data, Andraud et al. estimated the age at infection between 60.9 and 96.6 days [32]. Based on serological data from longitudinal studies in six pig herds, passive immunity was shown to delay early HEV infection of piglets by about 6 weeks in all but one farm on which the dynamics of infection were similar, whatever the animals’ initial serological status. Although the protective role of passive immunity cannot be denied, the latter case highlighted the strong interaction between farm-specific husbandry and hygiene practices and the HEV transmission process [32].

**Figure 1:**
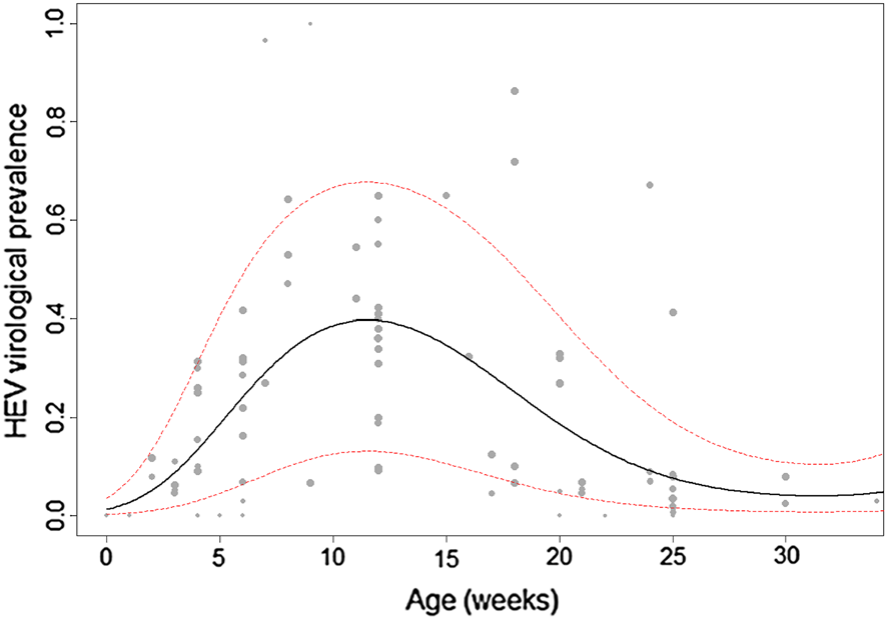
**Predicted HEV prevalence in faeces according to animal age.** The virological prevalence data (faecal shedding or presence in livers) depending on age (obtained from 31 published studies) were used to construct a meta-regression (generalised linear mixed-effect model) taking into account the respective weights of publications calculated using the inverse of the sum of inter-study and intra-study variance for a given age category. The mean predicted response of the model (black line) and its confidence interval (red dashed lines) are presented in this figure. The size of the points is proportional to the weight of the study.

HEV infection dynamics have in the majority of studies been described through the monitoring of shedding pigs. These studies showed that the prevalence of HEV RNA in swine faeces and serum depend on the production stage, i.e. the pig’s age (Additional file 2 [14, 15, 18, 21, 57, 66, 86–89, 91–93, 95, 96, 104, 108, 109, 113, 114, 118–120, 133, 135, 136, 140–150]). A broad shedding period from 1.5 to 5 months of age was globally reported at farm scale. In most cases, the faecal shedding peak was described in 3-month-old to 4-month-old pigs, and few animals had PCR-positive faecal samples after 6 months of age. We performed a meta-regression analysis using data from 31 studies published between 2002 and 2016 which reported the prevalence of faecal HEV shedding or presence of HEV in livers depending on pig age. A weighted generalised linear mixed-effect model with the publication as a random effect, using intra and inter-study variances for a given age category as weight for individual studies, was fitted to age-specific prevalence data. Despite marked variability between studies, the model showed that the probability of faecal shedding peaked around 90 days of age (Figure 1). The shedding prevalence estimate at 185 days (a common slaughter age) was 6.1% [1.2–15.4].

Few studies have explored the factors influencing variations in age at shedding. A longitudinal study conducted on three swine farms showed that HEV shedding in pigs which had been previously infected by Porcine Reproductive and Respiratory Syndrome Virus (PRRSV) occurred later than in pigs that were PRRSV-negative or which had been infected by PRRSV after HEV infection (hazard ratio = 0.49, *p* < 0.01) [33]. Hence, the latency period (delay between infection and shedding) may be affected by different circumstances, modifying the age at shedding. When pigs were infected intravenously, the latency period measured by Bouwknegt et al. was 3 days, compared to 6.9 days [5.82–7.9] when inoculated orally [34, 35]. According to the results of a co-infection trial, the HEV latency period was extended by a factor of 1.9 in the event of PRRSV co-infection (12.9 days [12.8–14.4]) [36]. In a recent longitudinal study on two Finnish farms identified as HEV-positive, piglets started to shed HEV between 7 and 12 weeks of age. Of these, 62.5% only began shedding HEV between the ages of 10 and 12 weeks [37]. The authors suspected that the difference in age of the onset of infection or shedding may have resulted from the quantity and/or quality of colostrum providing the piglets with maternal antibodies. The quantity of virus particles ingested may also modify the course of infection at the individual level. The minimal *per os* infectious dose is still not well known. However, it has been estimated that oral infection would require about 20 mg of faeces per day containing on average 10^8^ genome equivalent (GE) per gram over three consecutive days to achieve a 50% probability of infection [38]. These results were then confirmed by testing different infection doses for inoculation by the oral route: a minimal viral load of 10^6^ GE was necessary for pigs to be orally infected and to shed and transmit the virus [35]. Below that level, only sporadic shedding was detected, with no transmission to sentinel piglets.

#### Shedding duration and quantity of virus particles shed

Shedding duration is not easy to measure on farms as it requires an individual follow-up of pigs. The HEV shedding period was estimated at around 27 days in two studies carried out on commercial pig farms in Europe [33, 39]. Data from three pig herds evidenced a huge variation in the infection dynamics according to the farms, with some batches exhibiting late and short-term infections, while others had early and long-term shedding periods [31]. When pigs were experimentally infected by the intravenous route, shedding lasted from 13 to 49 days depending on the viral dose inoculated [34], whereas an HEV infection trial described a shedding duration of 9.7 days [8.2–11.2] when pigs were orally infected [35]. This experimental estimate was lower than shedding durations observed on farms, suggesting the existence of factors influencing duration of the shedding period. One of them may be other pathogens co-infecting pigs. For instance, one trial showed that a PRRSV/HEV co-infection dramatically extended the shedding period by a factor of 5 to 48.6 vs 9.7 days [36].

Few data are available on the quantity of virus particles shed by infected animals. In field conditions, one study carried out in two pig herds in Japan reported an HEV load in faeces of between 10^3.8^ and 10^6^ GE/g throughout the pigs’ life [40]. Similarly, the quantity of HEV particles shed was evaluated between 10^4^ and 10^6^ GE/g of faeces in an experimental trial involving 18 pigs that had been orally infected [35]. When pigs were co-infected with PRRSV, the viral load shed increased to between 10^5^ and 10^8^ GE/g of faeces, and the accumulation of HEV in the environment was significantly higher too [36].

#### Humoral immune response

Fourteen studies investigated the humoral response of pigs following HEV infection (Additional file 2). In a longitudinal study carried out on six Spanish farms [41], IgM antibodies were first detected at 7 weeks of age in five farms and at 13 weeks of age in only one farm, whereas IgG antibodies were firstly observed at 13 weeks of age in four farms and at 18 weeks of age in the two other farms. At slaughter age (26 weeks), IgG antibodies were detected in 50 to 100% of pigs on five out of the six farms. In the study conducted by de Deus et al. [42], IgG antibodies were detected later (around 15 weeks), whereas IgA and IgM appeared at around 12 weeks. Similarly to the previous study, IgG antibodies were detected up to the slaughter age (22 weeks), whereas IgA and IgM only remained for 4–7 weeks.

Thanks to field data collected in Japan, Satou and Nishiura estimated the time required for seroconversion at 25 days (95% CI 20.9, 31.3) [30]. Similarly, the time to HEV seroconversion was estimated at 26.3 days in an experimental study and it was shown that co-infection with PRRSV delayed the time to seroconversion to 43.1 days, increasing the lag to seroconversion by a factor of 1.6 [36]. The presence of maternal antibodies was also found to delay seroconversion in piglets [40, 43]. The time taken for maternally-derived antibodies to wane depends on the quantity of the initial intake of colostral antibodies [41], which is itself related to the sow’s age [44] and HEV immune status. Passively acquired IgG remained detectable up to 9 weeks of age in piglets born to highly HEV-seropositive sows, compared to 1–3 weeks in piglets born to sows with weak anti-HEV immunity [42].

#### HEV viraemia

The natural course of infection in pigs involves infection at around 8–12 weeks of age coinciding with declining maternal antibodies, with a generally short viraemia lasting from 1–2 weeks followed by a more prolonged period of viral shedding in the faeces (Additional file 2). However, longer viraemia periods have also been reported, especially in the case of late HEV infections in pigs, possibly lasting up to slaughter age (Additional file 2). In an experimental infection study, Sanford et al. observed more prolonged periods of viraemia in some pigs, and one pig was continuously viraemic for 12 weeks post-infection [45]. A study in Scotland showed that 44.4% of pigs tested were viraemic at slaughter age [16], whereas another study conducted in the UK only reported 3% of viraemic pigs at slaughter age [17]. Maternally-derived antibodies were found to delay the onset of viraemia [40]. Furthermore, the amount of HEV RNA in the serum was found to be lower than that in the faeces, the highest serum HEV RNA titre being on day 90 in a pig from a litter with passive immunity (10^4.2^ copies/mL) and on day 60 in a pig from a litter without passive immunity (10^5.6^ copies/mL).

### Routes of HEV transmission between pigs

It has been proved that the virus is mainly shed by the faecal route, leading to an accumulation of HEV in the pigs’ environment at all production stages on infected farms, as well as in manure pits [46]. Depending on the type of floor (litter or slatted floor), the animals are constantly in contact (more or less direct) with the environmental HEV reservoir. The virus has also been detected in the urine of HEV-infected pigs [38, 47, 48], making urine a potential transmission route, especially given the considerable volume produced per day and the potentially longer viral shedding in this medium [48]. Given the urinary and faecal shedding routes, drinking water and/or feed may also be indirect vectors of HEV transmission, especially if feeding and drinking equipment can be easily contaminated by faeces and urine [46]. Finally, daily repeated contacts between pigs kept in the same pen and housed in a confined environment, as well as the mingling of pigs at different production stages may increase the propagation of HEV on farms [23, 42, 49, 50]. These findings confirm that the faecal-oral route is the major transmission route of HEV in pigs [48, 49], even if several trials have highlighted the difficulty in inoculating pigs per os [51, 52]. Indirect transmission from one pen to another (without any pig mingling) was found to be low [35].

Three-month old and older pigs were recognised as the major shedding sources in farm conditions (Figure 1). Fernandez-Barredo et al. showed that weaning and early fattening stages were critical periods for HEV shedding with respectively 45 and 60% of shedding animals [46]. Breeding animals also play an important role in the spread and persistence of HEV within pig production units in two ways: (1) by providing maternally-derived antibodies that protect their piglets from early-life infection, (2) by possibly transmitting the virus via farrowing crates during lactation periods. Indeed, investigations into faecal shedding in sows around the farrowing period revealed prevalences ranging from 16 to 21% [41, 42, 46]. A high proportion of multiparous sows were found to shed the virus, as well as gilts and young sows but to a lesser extent [18]. A study recently carried out in China showed that farrowing sows had an approximately 2.5-fold higher risk of infection (OR 2.46, *p* < 0.01) than pre-farrowing sows [53]. Another study on Göttingen Minipigs in the context of xenotransplantation safety detected HEV in the sera of three sows 6 days after delivery and in their offspring [54]. Finally, in a longitudinal study on three pig farms, piglets from two farms shed the virus as early as the lactation phase in farrowing facilities [31]. Thus, horizontal transmission between sows and their piglets may occur in the early stage of a piglet’s life. Moreover, sows may transmit the virus to the foetus by the transplacentary route should viraemia occur during gestation, viral RNA having been detected in the livers of aborted fœtuses [55]. However, these results are still controversial, since one experimental study did not show any vertical transmission after intravenous inoculation of HEV to pregnant gilts [56]. Nevertheless, it cannot be excluded that breeding animals may constitute an HEV reservoir on infected farms, periodically shedding the virus according to changes in their immune status due to physiological conditions (pregnancy, farrowing). Sows may thus maintain viral propagation in swine herds.

### Quantitative data on HEV transmission

The persistence of a virus on farms is linked to (1) the intrinsic ability of the virus to remain in the animals’ environment, (2) the possibility of regular reintroductions of the virus onto farms and (3) the ability of the virus to survive and spread in the population. This last criterion can be studied through the basic reproduction number (R0) of the virus, which measures the number of secondary infections due to one infectious pig during its entire shedding period in a fully susceptible population. The higher the basic reproduction number, the easier it is for the virus to spread and the greater its ability to stay within the population. Using a large-scale seroprevalence survey dataset from Japanese pig farms, Satou and Nishiura estimated the HEV R0 between 4.02 and 5.17, meaning that one infectious animal could theoretically infect four to five other pigs during its infectious period [30]. Based on an experimental trial carried out in the Netherlands, this ratio was estimated at 8.8 [34]. However, this assessment relied on one-to-one HEV transmission experiments, accounting for horizontal transmission by direct contact only. The trial by Andraud et al. investigated the transmission of HEV from pigs inoculated by the oral route to pigs in direct contact (in the same pen) or indirect contact (in an adjacent pen) with the inoculated pigs, assuming both environmental and direct transmission routes [35]. Although much lower than previous estimates with a partial reproduction number of 1.41 [0.21–3.02], direct transmission alone could be considered as a factor fostering the infection’s persistence within a population. The quantity of virus present in the environment was found to play a pivotal role in the transmission process, strongly influencing the probability of infection, with a within-pen transmission rate estimated at 2.10^−6^ g/GE/day [1.10^−7^–7.10^−6^]. Between-pen environmental transmission occurred to a lesser extent (transmission rate: 7.10^−8^ g/GE/day [5.10^−9^– 3.10^−7^]) but could further generate a within-group infection process. The combination of these transmission routes could explain the persistence and high prevalence of HEV in pig populations. Moreover, the transmission of HEV was found even enhanced in the presence of co-infections. Indeed, based on a similar experimental design with pigs co-infected with PRRSV, the transmission of HEV by direct contact was estimated to be 4.7 times higher in pigs co-infected with PRRSV (direct transmission rate  =  0.70 [1.18.10^−3^–3.67]). Direct transmission therefore plays a more important role in HEV transmission when animals were co-infected and reflecting the increased quantity of virus particles shed [36]. The indirect transmission rate, considered to be the average number of animals that could be infected by a single genome equivalent present in the pen environment, was estimated at 6.59.10^−6^ *g*/GE/day [1.43.10^−10^–1.27.10^−4^], i.e. 3.3 times higher with co-infection than without. In other words, 3.3 times fewer virus particles were required to infect a co-infected animal than an HEV-only infected animal. The impact of maternally-derived antibodies on HEV transmission was also assessed by modelling field-based longitudinal data on HEV dynamics of infection [32]. In this study, HEV transmission among piglets with passive immunity was estimated to be 13 times lower than in fully susceptible animals, with a relatively marked variability between herds (range: 5–21).

## Consequences of HEV infection dynamics on the prevalence of contaminated livers and pork products

### Prevalence of HEV-containing livers at the slaughterhouse

In the ten studies investigating the prevalence of HEV-containing livers in pigs of slaughter age (Table 2), all but one reported prevalences ranging between 0.8 and 10% of liver samples, but the prevalence reported in Italy was over 20% [57]. Two conditions are required for a high prevalence of HEV-containing livers at slaughter age: (1) the virus has to spread massively on farms; (2) the later the infection occurred, the higher the risk that pigs are still infectious at slaughter. One study on French pig farms reported several risk factors, such as the slaughter age, genetic background, lack of hygiene measures and origin of drinking water [23]. An experimental trial also showed that the co-infection of pigs with HEV and PRRSV increased the likelihood of HEV-containing livers at slaughter time [36]. Satou and Nishiura built a model from field data and using a sensitivity analysis, they showed that a decline in the force of infection would postpone the infectious process to a later age, which would in turn heighten the risk of pork-to-human transmission through the consumption of infected products [30].

**Table 2:** **Prevalence of HEV RNA in livers collected at slaughterhouses reported in ten studies**

References	Country	No. of samples	Prevalence of RNA-positive livers (%) [95% CI]
Bouwknegt et al. [51]	Netherlands	62	6.5 [1.8–15.7]
Rose et al. [5]	France	3 715	4 [2–6]
Di Bartolo et al. [57]	Italy	48	20.8
Di Bartolo et al. [68]	Spain	39	3
	Italy	33	6
	Czech Republic	40	5
Berto et al. [67]	UK	40	3
Gardinali et al. [70]	Brazil	118	1.7
de Souza et al. [71]	Brazil	453	1.3
Temmam et al. [72]	Madagascar	250	1.2
de Paula et al. [73]	Cameroon	345	0.8
Mykytczuk et al. [74]	Canada	19	10.5

### Consequences on the safety of pork products entering the food chain

Nine prevalence studies were conducted on marketed pork products (Table 3). Different kinds of pork products were tested, such as raw livers, sausages, figatelli, pâté, etc. The prevalence of contaminated pork products varied from less than 1% to more than 50% depending on the country and the product. The highest prevalences were observed in products prepared with raw pork liver [7, 58]. No study was led on meat but, given the late viraemia at slaughter age that was observed in several studies (see above), there may be a potential risk to public health linked to the consumption of raw or undercooked pork meat.

**Table 3:** **Prevalence of HEV-positive marketed pork products reported in nine studies**

References	Country	No. of samples	Prevalence of RNA-positive pork products (%)
Yazaki et al. [75]	Japan	363	1.9% of livers sold in local grocery stores
Feagins et al. [76]	USA	127	11% of livers sold in local grocery stores
Colson et al. [7]	France	12	58% of marketed figatelli
Wenzel et al. [77]	Germany	200	4% of livers sold in butcher’s shops and grocery stores
Berto et al. [67]	UK	63	10% of marketed sausages
Di Bartolo et al. [68]	Spain	93	6% of marketed sausages
	Czech Republic	92	0% of sausages
	Italy	128	0% of sausages
Pavio et al. [58]	France	394	30% of figatelli, 29% of liver sausages, 25% of quenelles, 3% of dried salted livers
Heldt et al. [78]	Brazil	50	36% of marketed pâté and blood sausages
Mykytczuk et al. [74]	Canada	111	47% of pork pâté, 0% of raw pork sausages

The presence of HEV in food products consumed raw or undercooked raises the question of the thermal stability of HEV, which was addressed in three studies. The first one was based on heating faecal suspensions of HEV genotypes 1 and 2 to temperatures between 45 and 70 °C and inoculation in a cell culture permissive to HEV [59]. The second study used pigs inoculated with pork liver homogenates containing infectious genotype 3 HEV heated to 56 °C for 1 h, fried for 5 min (71 °C internal temperature) or boiled for 5 min [60]. Both studies showed that HEV was more likely to resist when heated to only 56 °C and was inactivated at temperatures higher than 71 °C. The third study was conducted on more complex foodstuffs prepared according to industrial recipes (liver pâté) and showed that it was necessary to heat the food to an internal temperature of 71 °C for 20 min to fully inactivate HEV [61]. To date, no information is available about the efficacy of drying on HEV persistence.

## Improving HEV surveillance and control in the swine reservoir: from farm-targeted actions to pork product control

### Options for control measures on pig farms

#### HEV vaccination on farms

No commercial vaccine is currently available against HEV in pigs. Some theoretical work has been carried out to evaluate the benefits of vaccination against this zoonosis, which does not have any consequences on pig health or the economic performance of swine herds. Using a modelling approach, Backer et al. tested three effects of vaccination: a decrease in the virus transmission rate, in animal susceptibility to HEV infection, and in the duration of the infectious period [39]. As previously shown by Satou and Nishiura [30], a reduced transmission rate and susceptibility, which induces a decrease in the force of infection, led to an increase in the number of infectious animals at the slaughterhouse. When the vaccine affected the duration of the infectious period, the proportion of pigs still infectious at slaughter age was lower. Further work would be needed to evaluate the required efficacy for a vaccine to eradicate the infection and to develop the corresponding efficient vaccine, without forgetting considerations on interference with passive immunity, co-infecting pathogens and rearing practices. A cost-benefit analysis of vaccine development would also be necessary, including public health consequences in the event of widespread consumer exposure to contaminated pork products, and the economic consequences linked to a potential loss of consumer confidence in pork safety.

#### Control of risk factors and co-infecting pathogens

As previously mentioned, a lack of hygiene measures and several farming practices (such as late weaning or mingling practices at the nursery stage) were reported as risky for HEV transmission and persistence. Biosecurity and farming practices should therefore be enhanced to reduce HEV risks [23].

As reported previously, co-infections with immunosuppressive swine viruses — frequently observed in pig herds — could lead to chronic HEV infection, which may dramatically increase the risk of pig livers containing HEV at slaughter time. For instance, a PRRSV/HEV co-infection or a PRRSV infection prior to HEV infection delayed HEV shedding and the onset of the anti-HEV humoral immune response, increased the quantity of virus particles shed and extended the shedding period, increased the direct transmission rate and HEV infection susceptibility, and increased the proportion of HEV-positive livers at slaughter time [33, 36]. Thus, controlling intercurrent swine diseases (e.g. through PRRSV vaccination programmes) could be a major lever in the control of hepatitis E. Further research is needed in this domain to better understand the interactions between HEV and immunosuppressive pathogens, including an evaluation of the effect of other immunosuppressive co-infections frequently encountered in the field as well as non-biotic components such as mycotoxins which are likely to interfere with the immune response.

#### Organisation of the pig production network

To prevent the spread of infectious agents, it is necessary to consider the pyramidal structure of the pig production sector and the way contacts between pig farms are organised [62]. Few data are available yet. A recent study reported the presence of HEV inside and outside farm buildings, on trucks and in the slaughterhouse yard, thus suggesting viral transmission between farms and throughout the production network [63]. However, further research is needed to (1) model the pig production network; (2) explain, assess and quantify the risk of HEV transmission between pig farms through animal introductions (replacement) or indirect vectors.

### Surveillance throughout the pork chain

To our knowledge, no uninterrupted surveillance programme of the swine reservoir has ever been implemented in any country. Surveillance actions could be implemented at different steps: on pigs at the farming stage or at the slaughterhouse, or on pig livers and pork products.

#### Monitoring of pigs on farms or at the slaughterhouse

Pig monitoring could be either serological or virological. (1) Serological monitoring could be a feasible large-scale approach. Data are available on the intrinsic features of the serological tests that could be used [64, 65], but further comparative analysis is still needed. Indeed, although a single HEV serotype exists, test performance varies depending on the HEV genotype [65]. However, more and more commercial ELISA tests are available and geared to HEV genotype 3, which is the main one circulating on pig farms in Europe and the US (e.g. HEV ELISA 4.0 V, MP Biomedicals). Moreover, some tests only detect IgM whereas others detect all immunoglobulin classes. Regarding the relevance of using serological tests, studies revealed a significant relationship between within-farm seroprevalence and the probability of detecting HEV-positive livers on that farm [5]. Indeed, Rose et al. observed that the probability of viral presence in the liver was significantly higher on farms where seroprevalence at the finishing stage was greater than 25%: OR 6.7 [2.1–21.6]. This result suggests that farms at risk are those in which the virus circulates intensely and spreads to more than 25% of fattening pigs [66]. However, at an individual level, some HEV RNA-positive pigs (detected in the liver) are seronegative because infection occurs late, not long before slaughter. This is why it appears that serological tests on fattening pigs from farrow-to-finish farms should be supplemented by tests on sows in order to clearly determine the HEV status of the farm. (2) The virus could also be detected in faeces as it appears that the virus’ presence in the liver and viral shedding are well correlated [31]. This surveillance action could be performed on farms, e.g. for a pre-slaughter check by sampling several animals. It could also be done at the slaughterhouse, in ante mortem waiting areas.

#### Surveillance of pig livers and pork products

Many human cases in industrialised countries are related to the consumption of so-called “high-risk” products, i.e. pork products consumed raw or not well cooked and containing a high proportion of pork liver. Surveillance could therefore target those specific products (liver sausages, liver pâté, figatelli, etc.). To date, few detection tests have been developed [61, 67, 68] and only one method for HEV detection in food has been marketed (HepatitisE@CeeramTools™, quantitative RT-PCR Kits for food & environmental samples). The viral concentration in food is often low. Moreover, these complex matrices are composed of liver, fat, salt and spices that make detection difficult. The analysis of meat matrices requires a rotary mill that is not frequently employed in non-specialised laboratories. Fat removal is essential, but tedious and mostly manual. Analysing food products is more complex than analysing livers, so livers could be tested after mixing and before adding other ingredients. As the transformation steps do not affect HEV stability (see above), the contamination of livers may be a relevant indicator of the risk of human exposure to HEV.

#### Applications and research needs

HEV monitoring activities in the pork production chain are needed for several purposes: (1) to acquire an uninterrupted series of prevalence data and monitor changes in prevalence and the virus itself (e.g. evolution of the prevalence of the different HEV subtypes and emergence of genotype 4, which is still only sporadically detected in Europe and the US); (2) to investigate more precisely HEV infection dynamics and factors influencing their variation; (3) to prevent contaminated livers from entering the food chain. For that purpose, the qualification of farms and/or animals and/or livers with regard to their HEV status could be considered. A French expert appraisal suggested three options that could be jointly implemented in order to prevent HEV-positive liver being used for the preparation of products containing raw liver [69]:

1. *Qualification of HEV*-*free farms* The farm could be qualified following serological testing on sampled animals (see above). This would enable the identification of farms eligible to market raw livers. However, this approach would be costly and would require constant testing, since the HEV status is unlikely to remain stable over time. Moreover, the logistics in slaughterhouses would then be complex, requiring an additional means of keeping HEV-free animals completely separate from HEV-positive ones.
2. *Real*-*time qualification of HEV*-*free batches at the slaughterhouse* Faecal samples could be taken from a determined number of pigs per batch, either in the ante mortem waiting area or the post mortem chain. The batches would be released after test results on a just-on-time basis. The HEV status of batches would be precisely known and only HEV-free batches would be used for the preparation of products containing raw liver. However, the logistics for the slaughterhouse would be both complicated and costly.
3. *Qualification of liver homogenates* RT-PCR could be performed on livers or liver homogenates to determine their HEV status. This approach would be less expensive yet would still enable an immediate risk management procedure to be followed depending on the result of the analysis. However, in the light of the HEV prevalence in livers, there would be a risk of detecting and rejecting many liver mixes.

These three options could lead to the creation of a separate sector dedicated to the fabrication of foodstuffs containing raw liver. Such a certification procedure requires regular food control capabilities relying on effective analytical tools for routine use, particularly on farms, at processing facilities and points of sale. The effectiveness of the certification system also relies on the traceability of pork livers, and requires a reference on the product label for all items containing pork liver. The label should provide consumers with information on the possible hazards related to consumption of these products. The coexistence of these two sectors may pose problems both in terms of logistics for the slaughterhouses and processing plants, and a risk of confusion for the consumer between products with different food safety statuses.

**Figure 2:**
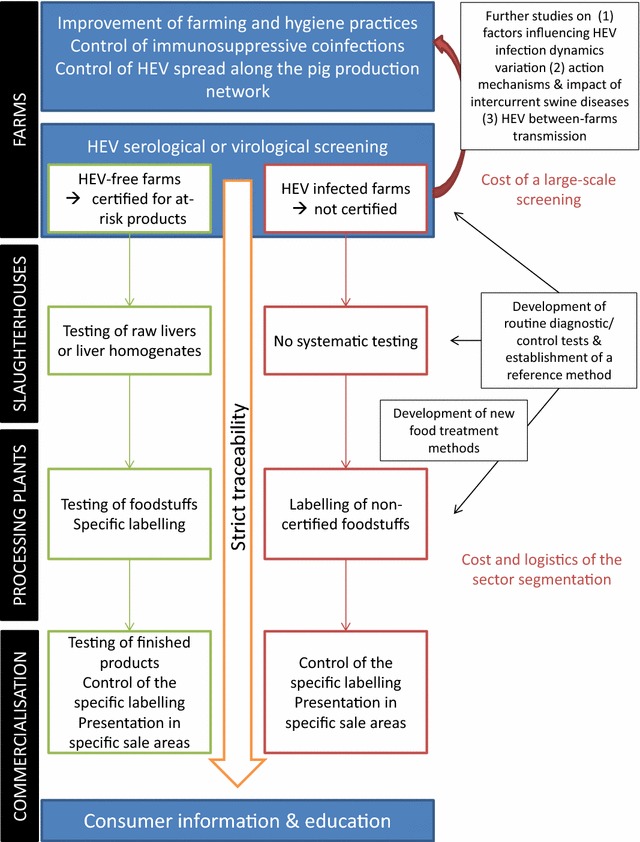
**HEV surveillance and control of the swine reservoir: from farm-targeted actions to pork product control (adapted from [** 69 **]).** The left side of the diagram presents a number of measures to mitigate the risk of human exposure to swine HEV, with actions applying to both farms and foodstuffs. A certification process (green and red squares) could be implemented throughout the food chain to guarantee the absence of HEV in products derived from raw pork liver. The right side lists several knowledge gaps and research needs (black squares) in addition to the challenges involved in implementing these measures (in red).

Whatever the qualification method, further studies are needed to compare the current tests, develop a reference method and establish a sampling plan geared to the sector’s situation. It is also necessary to investigate more precisely the risk linked to pork meat in order to assess the need for a meat surveillance and control plan. Figure 2 summarises the options for control measures throughout the food chain, and identifies knowledge gaps and challenges.

## Conclusion

The epidemiology of HEV in the swine reservoir is far from being fully elucidated. Though many prevalence studies have been carried out in numerous countries in the last decade, there remain knowledge gaps that still have to be addressed. Research needs to focus on the factors that could explain the huge between-herd variation in infection dynamics, HEV transmission between farms and throughout the pig production network, and finally the mechanisms of action and impact of intercurrent swine diseases. Further work also needs to be carried out to harmonise diagnostic tests and develop a standard reference method to detect HEV in complex foodstuffs. Surveillance plans and control programmes have to be carefully considered to mitigate the risk of human exposure to HEV through the consumption of pork products.

## Additional files

**Additional file 1.** **HEV serological and virological prevalence in the pig population at farm and individual levels.** Farm-scale prevalence figures were reported in 34 studies. Farm-scale seroprevalence ranged from 30 to 98%, while farm-scale virological prevalence ranged from 10 to 100%. Individual prevalence figures were reported in 82 studies. Individual seroprevalence ranged from 8 to 93%, whereas individual virological prevalence ranged from 1 to 89%.

**Additional file 2.** **Evolution of HEV RNA prevalence and anti-HEV antibody prevalence according to pig age.** Thirty-seven studies explored the variation of HEV virological and serological prevalence with the pig age. m: months; w: weeks; d: days.
